# Worth Your Knowing

**Published:** 1895-07

**Authors:** 


### WORTH YOUR KNOWING.

A daily ration for a camel of good size consists of about twenty pounds of dry or green fodder, with about eight pounds of barley flour, the latter made into a paste ball and rammed into the throat. He will carry a burden of 400 pounds and go without water four or five days.

Anthrax has broken out to quite an extensive degree in the grazing districts of Colorado, following the many heavy rains which have prevailed there.

The Philadelphia Milk Inspectors report for the month of June 49,885 quarts of milk inspected; 1360 of this amount condemned as watered, skimmed, or colored.

DISEASES of camels are said to be little understood.

